# Non-Destructive Thermography Analysis of Impact Damage on Large-Scale CFRP Automotive Parts

**DOI:** 10.3390/ma7010413

**Published:** 2014-01-14

**Authors:** Alexander Maier, Roland Schmidt, Beate Oswald-Tranta, Ralf Schledjewski

**Affiliations:** 1 Chair of Processing of Composites, Montanuniversität Leoben, Otto Gloeckel-Strasse 2, Leoben 8700, Austria; E-Mail: ralf.schledjewski@unileoben.ac.at; 2 Chair of Automation, Montanuniversität Leoben, Peter-Tunnerstraße 27, Leoben 8700, Austria; E-Mails: roland.schmidt@unileoben.ac.at (R.S.); beate.oswald@unileoben.ac.at (B.O.-T.); 3 Christian Doppler Laboratory for Highly Efficient Composite Processing, Montanuniversität Leoben, Otto Gloeckel-Strasse 2, Leoben 8700, Austria

**Keywords:** non-destructive testing, thermography analysis, impact damage behavior, impact test, structural automotive parts

## Abstract

Laminated composites are increasingly used in aeronautics and the wind energy industry, as well as in the automotive industry. In these applications, the construction and processing need to fulfill the highest requirements regarding weight and mechanical properties. Environmental issues, like fuel consumption and CO_2_-footprint, set new challenges in producing lightweight parts that meet the highly monitored standards for these branches. In the automotive industry, one main aspect of construction is the impact behavior of structural parts. To verify the quality of parts made from composite materials with little effort, cost and time, non-destructive test methods are increasingly used. A highly recommended non-destructive testing method is thermography analysis. In this work, a prototype for a car’s base plate was produced by using vacuum infusion. For research work, testing specimens were produced with the same multi-layer build up as the prototypes. These specimens were charged with defined loads in impact tests to simulate the effect of stone chips. Afterwards, the impacted specimens were investigated with thermography analysis. The research results in that work will help to understand the possible fields of application and the usage of thermography analysis as the first quick and economic failure detection method for automotive parts.

## 1. Introduction

Starting its development as an aerospace material, fiber-reinforced and particle-reinforced composites have been increasingly used in other industries, for example automobiles, marine transport, buildings and civil infrastructure, sporting goods, medical equipment and prosthetic devices, *etc*. With the increased use of composite materials, there is a tremendous need to develop efficient manufacturing techniques, economical and effective repair techniques and methods to predict the short- and long-term behavior of the composite materials and structures made of these materials under a variety of loading and environmental conditions. In addition to the aforementioned requirements, the construction and processing need to fulfill the highest requirements in the weight and mechanical properties [1].

Especially for composite parts in automotive applications, the environmental issues, such as fuel consumption and exhaust emissions, set new challenges in producing lightweight parts that meet the highly monitored standards for these branches. In the automotive industry, one main aspect of construction is the impact behavior of structural parts [2,3].

Fiber reinforced composites have tremendous strength and stiffness in the plane and main direction of the fibers. However, their strength and stiffness perpendicular to the fibers is governed mainly by matrix properties. Thus, composite structures perform poorly under impact loads that occur due to dropped hand tools, runway debris and hailstone. On the one hand, it is important to understand the threshold impact energy that will cause impact damage and, also, the residual strength after impact damage has occurred, and on the other hand, the detection of impact failure through non-destructive testing methods is necessary. Furthermore, testing the quality of newly designed parts at low cost and time is crucial in competitive fields, such as the automotive field. Therefore, non-destructive test methods are increasingly used [3–5]. As a matter of fact and as confirmed by several independent research works, ultra-sonic testing equipment provides the most information for high quality impact characterization [6–8]. Due to the rising dimension of composite applications, for example rotor plates of wind energy applications or huge structural parts for airplanes and automobiles, it becomes more and more important to find a quick and economic way to detect failures due to impact, which are sometimes barely visible, but nevertheless compromise the composites efficiency. Therefore, non-destructive thermography analysis gives us the ability to do a quick area detection on these parts and, in a further step, using ultrasonic analysis for single-point detection, to gather more detailed information about the amount of damage.

## 2. Background

### 2.1. Vacuum Infusion

Basic vacuum infusion is an easy method for manufacturing endless fiber reinforced composite parts. Therefore, only a simple, geometry-defining mold, vacuum bag, vacuum pump, resin and reinforcements are needed. In this work, for test specimen production, a slightly modified vacuum infusion was used. The slightly modified concept of vacuum infusion is to spread the resin in large areas by the help of flowing agents and to impregnate the reinforcements through the thickness in a manner more similar to the method description for the Seaman Composites Resin Infusion Molding Process (SCRIMP). The benefit of this mechanism is that the resin has only a short infusion path through the thickness of the manufactured parts, rather than a long way through the length of the parts. The maximum possible diameters (length, width and height) are dependent on the flow-resistance and the type of resin, as well as the reinforcement structures, the component thickness and the effectiveness of the vacuum [1,9,10].

**Figure 1.**
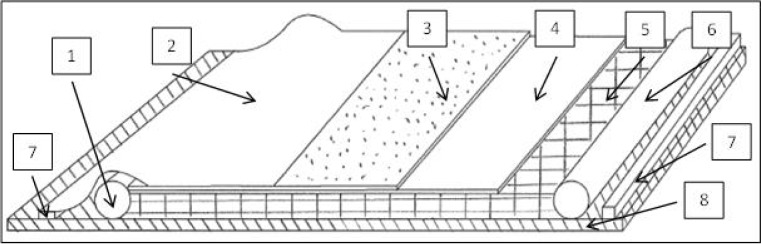
Schematic drawing of a typical vacuum infusion construction; mold assembly: 1, inlet; 2, vacuum bag; 3, distribution medium; 4, peel ply; 5, reinforcement; 6, outlet; 7, vacuum seal; 8, mold die; referring to Correia N.C. *et al*. (2005) [12].

The basic requirement for vacuum infusion is to guarantee that the mold used and the vacuum infusion structure are as airtight as possible. If this precondition of generating an effective vacuum is not fulfilled, the result will be shown in poorly manufactured parts, e.g., parts that include dry spots and/or entrapped air. Inside that area, the reinforcements are placed in order of the possible variety of loadings that they have to resist. In some cases, to improve the laying down of the enforcement parts, it is possible to use a binder to fix the dry reinforcements on the contour of the mold or to use other preform techniques, for example, like stitching and cutting, net-shape preforming or direct fiber. Normally, with vacuum infusion, composite parts with a fiber percentage up to 60% are possible [11]. As the next step, a resin feed wire and a suction wire have to be placed correctly in the mold to guarantee quick and proper resin infusion and impregnation. As an additional chance to improve the results of the vacuum infusion, simulating the infusion process provides extra information and a possible chance to rethink the whole procedure. On top of this construction, a peel ply, flowing agents, resin absorption material for spare resin and the vacuum bag are the last applied components. After finishing the construction, a vacuum test is necessary to check if the whole mold is airtight, and in the case that the test is positive, completing the impregnation can be started by filling the part with resin by using the suction power of the vacuum. Subsequent to the filling and impregnation process, the resin has to cure, and the manufacturing is done [12]. In the following a schematic drawing, a typical vacuum infusion construction is shown (Figure 1).

### 2.2. Impact Test

**Figure 2.**
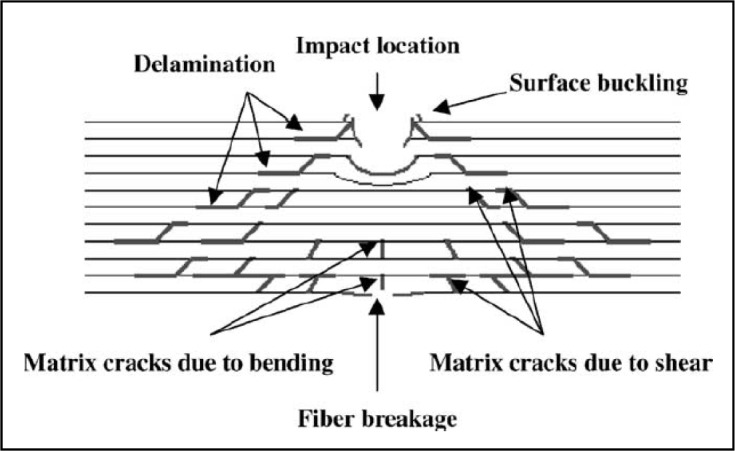
Schematic representation showing a typical impact damage mode for a composite laminate; referring to Tien-Wei S., Yu-Hao P. (2003) [26].

Impact energies can induce complicated forms of damage, for example, as shown in Figure 2. Damage in composites often begins on the non-impact surface or in the form of an internal delamination, and often, the detection and characterization of impact damages are difficult. The impact damage mechanism in a laminate constitutes a very complex process. It is a combination of matrix cracking, surface buckling, delamination, fiber shear-out and fiber fracture, *etc.*, which usually all interact with each other. The delamination caused by the mismatching of the bending stiffness was propagated and aligned along the direction of the fibers [1,13,14]. The delamination pattern is dependent upon the structure of the fabric. It requires an understanding of the basic mechanics and the damage mechanism [15]. As a matter of fact, composites can fail in a wide variety of modes, because nearly every impact damage mechanism reduces the structural integrity of the component. Even barely visible impact damage can cause fatal destruction to the composite parts, because most composite parts are brittle and, therefore, can only absorb energy in an elastic mechanism or damage mechanism [16]. Impact behavior is even more difficult to understand in applications where hybrid composites are used. In many advanced engineering applications, such as the automotive and aviation industries, hybrid composites have been extensively used due to their high strength, low weight, good fatigue life and corrosion resistance. In addition, their behavior under impact loading has been of significant concern in engineering. There are many studies in the literature [17–22] of the impact response of composite materials and structures. For example, Ying [23] has investigated the damage resistance of three types of carbon laminates and fabric reinforced composites and used finite element analysis coupled with a failure technique to predict the impact threshold energy of the laminates. Low velocity impact damage to composite parts, most of the time, are due to both operational and maintenance activities. In the operational environment, there are typically few incidents of low velocity impact damage, and most can be attributed to hailstone strikes and foreign object damage, such as runway debris, e.g., stone thrown by the tires. The major source of low velocity impact damage for structural composite parts is due to mishandling and maintenance mishaps that include part transportation, handling and storage, and incidental tool drops. However, the impact energy is initially absorbed through elastic deformation till a threshold energy value. Beyond this value, impact energy is absorbed through both elastic deformation and the creation of damage through various failure modes. The type of failure depends on the material and geometric properties of both the impactor and target materials. It has been documented by several investigators that damage initiation is manifested in the load-time history as a sudden load drop due to the loss of stiffness from unstable damage development. Subsequently, damage growth will arrest; the composite laminate will reload, and a cycle of damage propagation and arrest occurs until the impactor begins to rebound and the laminate is unloaded [24,25]. However, damage in composites often begins on the non-impacted surface or in the form of an internal delamination. To gather more information about the damage profile caused by an impact test, many testing procedures are used. Several inspection techniques (acoustic emission, thermography, dye penetrant, stereo X-ray radiography, ultrasonic), with different sensitivity levels, can be used for the non-destructive evaluation of composite materials. All these testing procedures are divided mainly into area detection, e.g., thermography analysis and single-point detection, e.g., ultrasonic analysis. Many investigators have studied the empirical relationship between the initial kinetic energy of the impact projectile and the area of the damaged region detected by mainly single-point detection, like ultrasonic analysis. However, in the case of barely or not visible low impact damages, single-point detection methods are stretching their limits, because screening a large area is not economic and requires too much time. In that case, detecting the damages is part of the area detection, like thermography analysis, and ultrasonic analysis is a part of further analysis to get more information about well-known damage spots [6–8].

## 3. Thermography Analysis

In the case of active thermography inspection, in the first step, heating is applied to the tested specimen, and with an infrared camera, the spatial and temporal distribution of the surface temperature is recorded. As cracks, defects and delaminations disturb the heat flow, evaluating the infrared images can reveal hidden failures. Heating the sample can be carried out optically, e.g., with a short flash pulse, with a laser or with halogen lamps [27]. To electrical conductive materials, such as metals or CFRP (carbon fibre reinforced polymers), heat can be very effectively applied by induced eddy currents. In the case of ultrasound stimulated thermography, cracks are selectively heated by the elastic waves [28]. In many cases, the heat is applied in a short pulse form, but in other cases, it can be sinusoidal modulated to use the so-called lock-in technique [29]. Additionally, in the last few years, different techniques have been developed for how the temporal change of the temperature can be evaluated, in order to obtain high contrast images of subsurface defects [30].

The answer to a short heating pulse, as from a flash lamp, can be evaluated in the frequency domain, with the so-called Pulse Phase Thermography (PPT) [31]. Fourier transformation of the temporal change of the temperature in each pixel of the infrared image sequence defines complex thermal waves. Usually, the phase values of these waves are investigated. As thermal waves with different frequencies penetrate into different depths of the material, phase images belonging to different frequencies exhibit failures in different depths [31].

Another possibility to evaluate in the time domain of the response to a short heating pulse is to calculate first or second derivatives of the temporal temperature change. These make the heat flow and the change of the heat flow visible. However, in order to calculate the derivatives of measured data, as a preliminary step, a polygon fitting is necessary to reduce the noise. This polygon fitting, called Thermographic Signal Reconstruction (TSR) [32], is carried out in the double-logarithmical scale, and it also allows for a very good reconstruction of the images with a low noise level [32].

It has been also shown [27–33] that, first, using a TSR technique and, then, calculating the phase images with Fourier transformation significantly enhances the signal-to-noise ratio of the images and, therefore, increases the detectability of defects deep below the surface.

**Figure 3.**
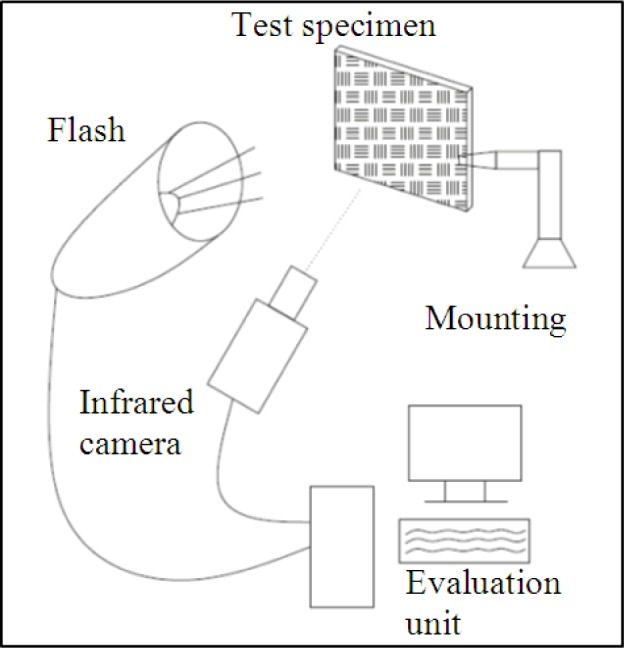
Thermography analysis testing setup.

In our experiments, a flash lamp was used with a 1 ms (full-width half-maximum) heating pulse with an energy of 6 kJ from one xenon flash lamp at a distance of 60 cm from the specimen. The heated side of the CFRP samples was imaged with a commercially available cooled 320 × 256 pixel InSb (indium-antimony detectors) camera, operating at 385 Hz in the 1.5–5 μm spectral range (Figure 3).

## 4. Test Specimen Preparation

### Production of CFRP Testing Specimen

The composite plates were manufactured from 0°/90° twill woven carbon fabrics (style 442 3K Aero) and epoxy resin by the vacuum infusion method. The epoxy resin used was EPIKOTE™ Resin MGS^®^ RIMR 135, and the hardener was EPIKOTE™ Curing Agent MGS^®^ RIMH 135, before the manufacturing process, stored at room temperature. The mixing ratio for resin-to-hardener in weight was 10:3. The composite plates were cured for 24 h at room temperature (23 °C) and under vacuum at a constant−900 mbar pressure.

**Figure 4.**
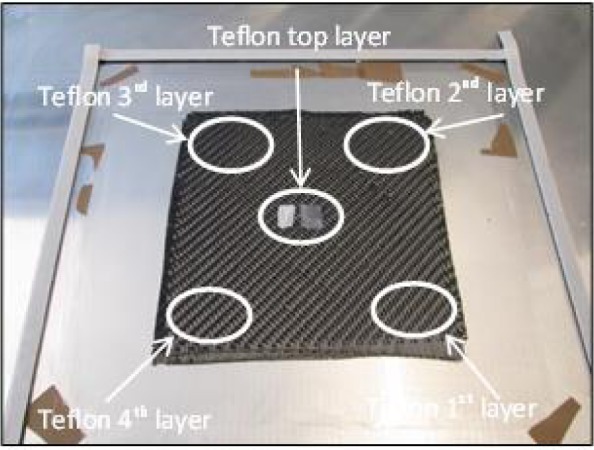
Test specimen, first thermography analysis.

For the first thermography analysis, the testing specimens were produced with dimensions of 400 mm × 400 mm, 5 layers, and between each layer, small Teflon pieces of 10 mm × 10 mm were positioned to simulate delamination (Figure 4). Due to this stacking, a thickness of the testing specimen of 3.2 mm was reached.

For the second part of this work, stacking sequences for the test specimen for the impact tests of (C_0_/C_90_)-woven at 45° + (C_0_/C_90_)-woven at 0° and, again, (C_0_/C_90_)-woven fabrics at 45° were chosen. This layer configuration of the composite plates was given due to the operating condition of a real automotive part produced in the Cars Ultra-light Technology project (CULT). After the manufacturing process, the composite specimens with dimensions of 150 mm × 100 mm were trimmed from the laminated plates.

## 5. Impact Testing

In this work, all low velocity impact tests were based on the ÖNORM EN ISO 6603:2 [34,35], and an Instron-CEAST 9350 impact testing machine (Instron GmbH, Pfungstadt, Germany) was used for impact testing. This testing machine consists of a dropping crosshead with its accessories, a pneumatic clamping fixture, a pneumatic rebound brake and an impulse data acquisition system. The weight of the crosshead is adjustable with the drop mass, and the tup of the impactor has a 20.0 mm diameter hemispherical nose. The self-identifying load-cell capacity is 15.56 kN, and the total mass of the impactor with its accessories was kept constant at 2.045 kg for all tests. The test machine has a pneumatic rebound brake system to prevent repeated impacts on specimens. CEAST View 5.94 3C is a software program that records the electronic signals (load *vs*. time data and instantaneous velocity at the moment of impact). The software program, based on Newton’s second law and kinematics, is used to convert the load-time data into a load-deflection relation, with the assumption that the impactor is rigid. The electronic signals are used by the software to calculate the deflection, tup velocity and the energy absorbed by the specimen. For the conducted impact tests, the impact energies varied from approximately 1 J to 5 J or even up to the complete perforation of the specimens. Therefore, it becomes possible to examine the damage mechanisms of composite plates under various impact energies.

**Figure 5.**
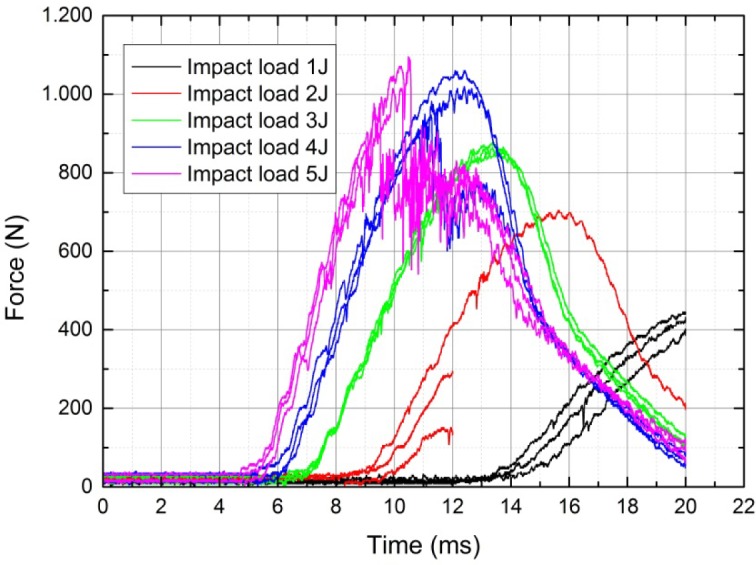
Impact result on the carbon test specimen for 1 J to 5 J.

As shown in Figure 5, it was not possible to detect matrix cracking, surface buckling, fiber shear-out and fiber fracture from the impact results for the impact loads of 1 J to 2 J. The test series under a 1 J and 2 J impact load showed no typical impact response. As a reason for these responses, we found out that the resolution of the testing machine was not great enough for measuring the impact at such low impact energies. Therefore, for the impact tests of 1 J and 2 J, no further evaluations were possible, and the testing series were not used for further analysis. For the impact results for the impact load of 3 J, it was not possible to detect matrix cracking, surface buckling, fiber shear-out and fiber fracture either, but the testing resolution was high enough, and therefore, we decided to use the impact results for further analysis. For all tests at 4 J and 5 J, at least one aforementioned mechanics failure was detected. For all of the impact tests from 3 J to 5 J, delamination between any of the layers was possible, and therefore, the thermography analysis came in handy. The capabilities of this non-destructive testing method are shown in this work.

## 6. Results of the Thermography Analysis of Teflon Tests

**Figure 6.**
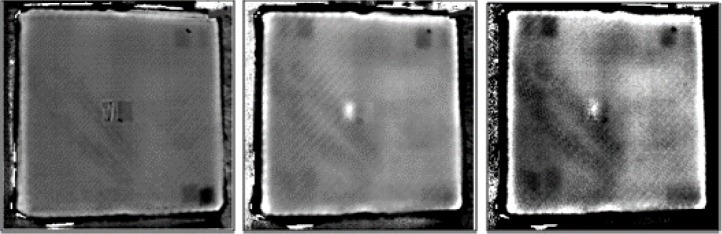
Phase images, after using Thermographic Signal Reconstruction (TSR), of the sample with Teflon inserts, shown in Figure 4. The frequencies of the images are 0.1 Hz (**left**), 0.15 Hz (**middle**) and 0.18 Hz (**right**).

The advantages of the thermography testing are that not only the subsurface defects can be made visible, but also, according to the delay of the response, their depths can be estimated. Teflon inserts are often used in CFRP structures to create artificial delaminations. A specimen, as shown in Figure 3, has been prepared with five layers and with Teflon inserts between them. The temperature response to a flash pulse heating has been evaluated by the PPT technique, following a TSR step, to increase the signal-to-noise ratio of the images. Figure 6 shows the results, and the figures from left to right demonstrate that with the evaluation of different frequencies, it is possible to make deeper and deeper images of the delaminations.

## 7. Results and Discussion Thermography Analysis/Impact Tests

### 7.1. Load/time Impact Result (F/t–Diagram)

**Figure 7.**
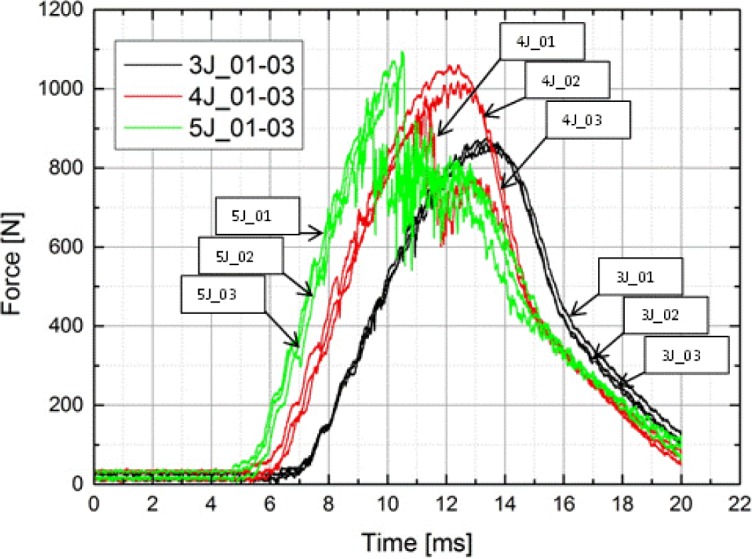
Load/time (F/t) curves of the carbon specimen for thermography analysis.

Figure 7 shows the Load-time (F/t) curves of the carbon testing specimens for thermography analysis at different levels of impact energy as an extract of Figure 5, because in this study, the results will be based on these impact energies. Individually, each curve has an ascending section of loading, reached a maximum load value and has a descending section of unloading. The ascending section of the Load/time curve is called the bending stiffness, due to the resistance of the composite to impact loading, and at this section, the maximum load value reached the highest maximum load; this value is called the peak force.

Typical Load/time curves of composite plates subjected to impact loading have three situations, including rebounding, penetration and perforation. Furthermore, load-time curves, in general, can be classified as closed-type curve and open-type curve. The rebounding case results in closed curves, indicating the rebounding of the impactor from the specimen surface. The closed-type curves return back from the maximum load or the peak force value towards the axis of abscissas means to 0 N force without a sudden load drop due to the loss of stiffness from unstable damage development. These curves are all curves with 3 J impact energy (3J_01–03), and, at an impact energy of 4 J, the second and the third one (4J_02, 03) are as seen in Figure 7.

When the impact energy increases, closed-type curves bound larger areas, and deflection increases while the rebounding section becomes smaller. As seen from Figure 7, at an impact energy of 4 J, there are closed-type (4J_02, 03) curves, but also open-type curves (4J_01). From that point on, as the impact energy continuously increases, the curve type changes from the closed-type to the open one. If a curve is of an open type, the specimen is either penetrated or perforated by the impactor. Therefore, the testing specimen at 5 J (5J_01–03) represents the penetrated case, while the others represent the perforated case, as seen in Figure 7.

### 7.2. Optical Impact Characterization and Thermography Analysis

The damage profiles of damaged composite specimens were evaluated from the front (impacted) and back (non-impacted) side by visual inspection and thermography analysis. In general, impact damage modes consist of indentation, matrix cracking, delamination between layers, fiber pull-out and fiber breakages. Impact-induced damage, which may be undetectable by visual inspection, can have a significant effect on the strength, durability and stability of the composite structure. Again, due to the fact that this work is about the detection of failure in general, there is no further examination of which type of failure is detected after the detection itself. In the following paragraphs, for understanding the possibilities and importance of non-destructive thermography analysis with respect to the possibility of doing quick and keen area failure detection, several images of damaged specimens were compared and explained with the load-time curves, visual detection and thermography analysis results. Therefore, the carbon testing specimens at impact energies of 3 J, 4 J and 5 J were used.

**Figure 8.**
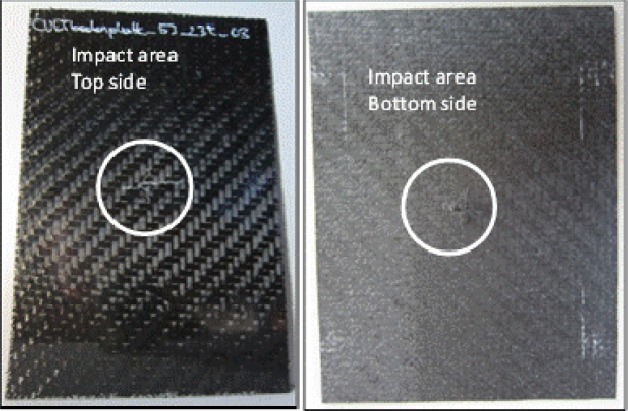
Visual failure detection for an impact energy of 5 J; (**left**) top side up; and (**right**) bottom side up.

**Figure 9.**
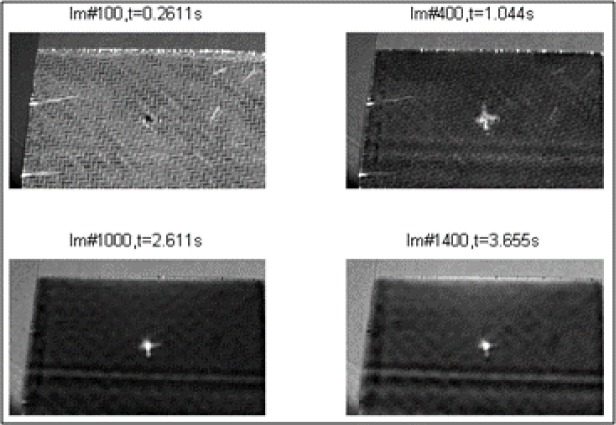
Catastrophic damage of the specimen shown by the thermography analysis after impact testing at 5 J, the second derivative of TSR.

At the highest impact energy during this work (5J_01-03), penetration and perforation started to take place for the carbon testing specimen, as shown in Figure 7. The penetration threshold can be defined as the energy level when the impactor gets stuck in the specimen for the first time and does not rebound from the specimen surface any more. The perforation threshold can be defined as the energy level at which the impactor passes through the thickness of the specimen for the first time resulting in permanent catastrophic damage to the specimen. As seen in the visual inspection (Figure 8), all carbon fibers are damaged through the thickness, but compared to the F/t curves, the energy was not enough for the impactor to get stuck in the specimen; a short rebound is seen in Figure 7 for the 5 J tests. Due to the visual inspection, the perforation process started at this point, but a complete perforation (catastrophic damage) was not seen. These results from the impact test and the visual detection are congruent with the result given by the thermography analysis, as seen in Figure 9. As additional information, the bottom pictures in Figure 9 showed two horizontal line marks on the specimen, which refer to the placement of the flowing agent during the vacuum manufacturing. These marks came from the vacuum under pressure and are due to the compaction of the rims of the flowing agent being marked on the composite plates. That result is not a part of this study and, therefore, not evaluated further. The same applies to Figures 11 and 12. However, the thermography analysis displays the damage section (fiber breakage, penetration, lamination, *etc.*) perfectly as a cross in the center of the impact zone. The four pictures at different times show the damage profile through the specimen thickness and gives a good overview that the impact damage goes through the whole testing piece, and as a conclusion, the thermography analysis is comparable with the F/t curves from the impact tests, therefore, for this stage of damage, the possibility for non-destructive damage detection.

**Figure 10.**
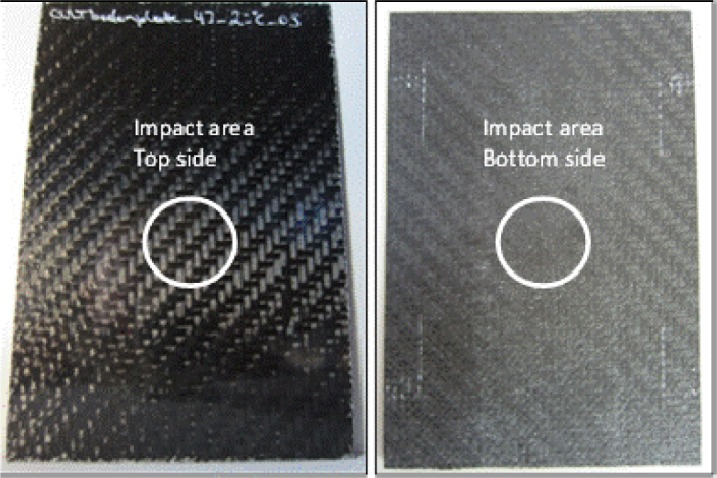
Visual failure detection for an impact energy of 4 J; (**left**) top side up; (**right**) bottom side up.

**Figure 11.**
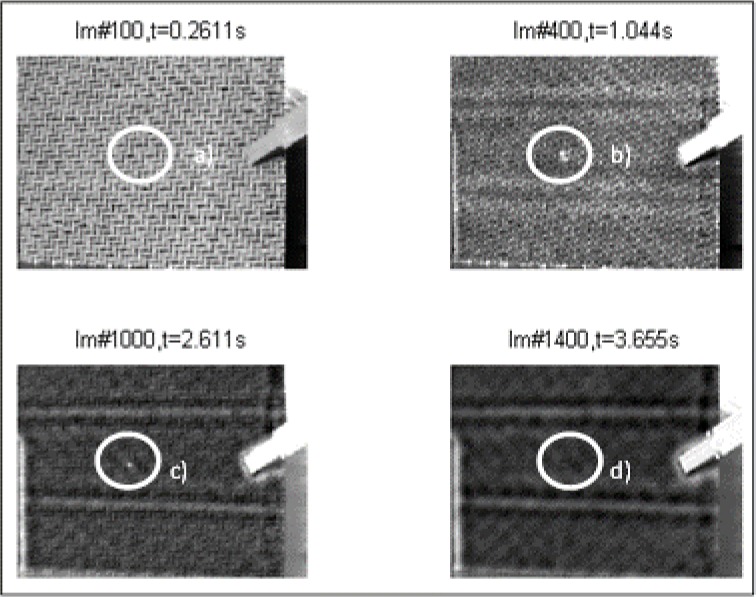
Thermography analysis after impact testing at 4 J, the second derivative of TSR.

As the impact energy decreases (4 J), the F/t curves of composites shrink in the negative direction of the horizontal axis, and due to the fact that the damaged composites had to absorb less impact energy, the damage profile of the catastrophic damage picture does not immediately show failure, as expected. F/t curves for 4 J compared with the visual inspection show the same tendency of lesser damage at lower impact energies, except for the testing specimen, 4J_03. These impact tests showed an open F/t curve, and therefore, the beginning of penetration and perforation would be expected. In Figure 10, as seen in the sectioning of the damage, there are no fiber breakages or penetration through the thickness of the carbon layers. The visual inspection of the other 4 J test specimen (4J_01, 02) showed the same result as the specimen, 4J_03. There are only small scratches, and on closer examination, matrix cracking and impact signs (small dots) of the impactor were shown, as seen in Figure 10. Regarding the F/t curves, it seemed that around an impact energy of 4 J, the damage profile is in a transition area between catastrophic damage and possible hidden damage inside the carbon layers. Therefore, heavy damage inside the specimen is possible, but with the F/t curves and the visual inspection, not verified. In part (a) of Figure 11, the thermography analysis showed no damage sign, and due to that very first image, it seemed like the same result as the F/t curves and the visual inspection. Analysis through the thickness of the test specimen thermography pictures (b) and (c) (more time leads to the inner carbon layer) showed damage, seen in Figure 11, as a highlighted spot. That spot means that between the layers, there is delamination caused by the impact test before. In that case, the non-destructive thermography analysis gave the possibility of detecting existing damages inside the specimen, which were not detectable with conventional testing methods. Going on to the last part (d), the thermography did not detect damage at all, and as a conclusion, and compared to the visual detection, the test specimen had no damaged top or bottom layer; only delamination between layers occurred.

**Figure 12.**
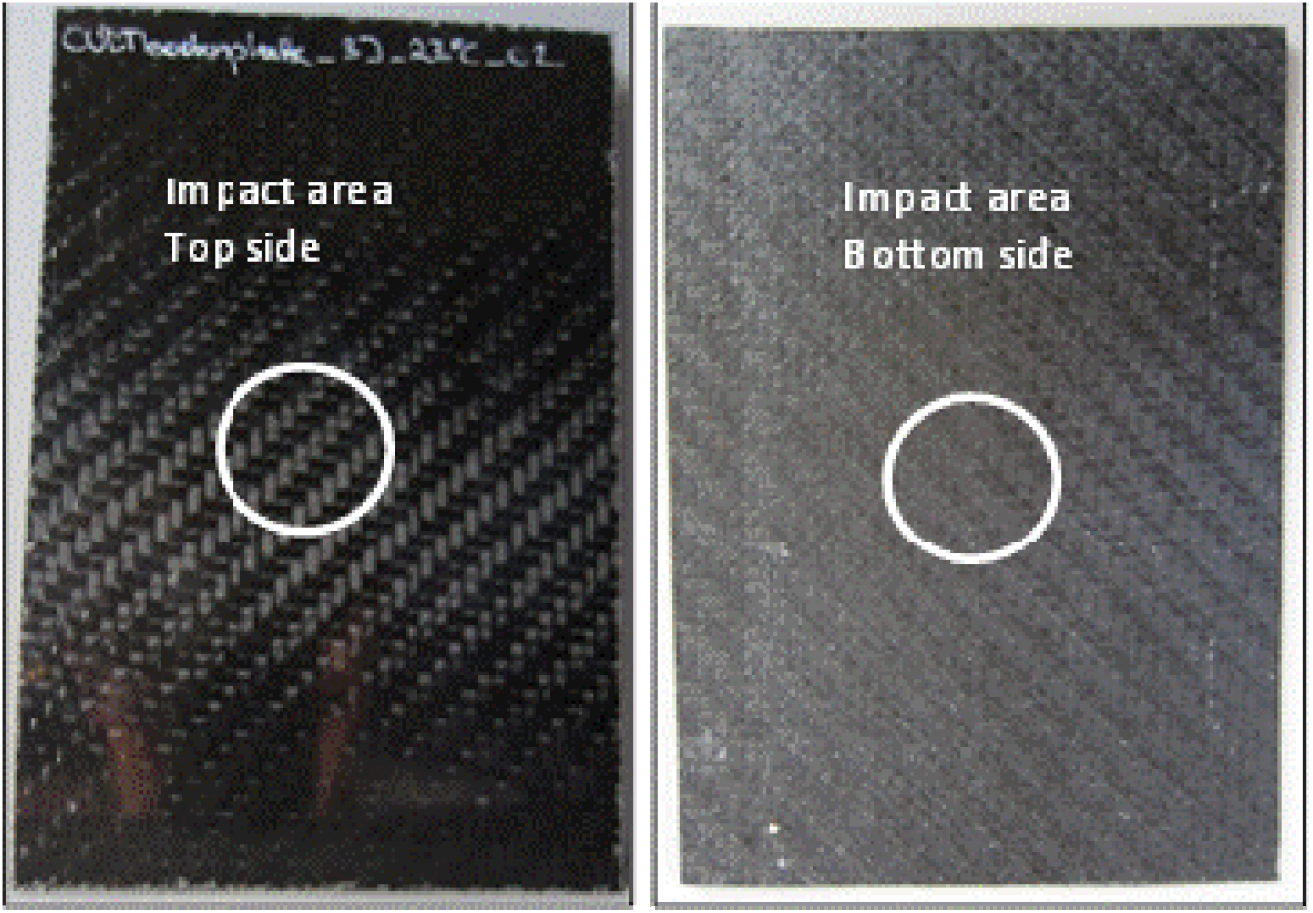
Visual failure detection for an impact energy of 3 J; (**left**) top side up; and (**right**) bottom side up.

For lower impact energies (less than approximately 3 J), the primary damage mode was indentation-induced matrix cracking on impacted surfaces. There were minor matrix cracks on the bottom side of the test specimen, and some delamination could be possible between the inner layers (Figure 12). As shown in Figure 7 (3J_01–03), the impact curve is a closed one, and compared with the visual detection of the top side up or the bottom side up (Figure 12), there is no penetration and/or perforation. As a result of these analyses, there are no failures detected. Nevertheless, interior layer delamination could be possible, but was not detected with the mentioned methods.

**Figure 13.**
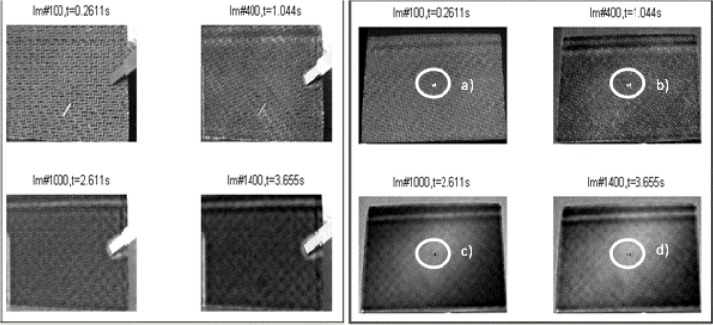
Thermography analysis after impact testing at 3 J; (left) top side up; and (right) bottom side up; the second derivative of TSR.

At that point, the degree of resolution of the thermography analysis provides, once again, further information of the failure profile of this testing specimen, shown in Figure 13. With the thermography analysis, a small failure crack at the bottom side of specimen was found. In fact, that there is no sudden drop in the F/t curve from the impact test right after peak force, which would detect fiber breakage in interior layers or perforation/penetration, none of these failures were expected. Referring to the visual inspection, the little black dot shown in the right pictures of Figure 13, there is no matrix cracking. One possibility was that this failure could be a delamination between the layers. In Figure 13, the right side, the different parts (a–d) show images along time, and therefore, it could be possible to provide information about the horizontal failure spread. However, for this impact energy, separated from the other analysis results, the non-destructive thermography analysis showed that there is a failure mark due to the impact. If this specimen were a real carbon composite part in a car, as the next step, the impact area, especially, and the area around it should be further inspected, and based on that, a statement about whether this part could be used further on could be made.

## 8. Conclusions

This experimental study deals with the investigation of impact damage composite plates using non-destructive thermography analysis to show the ability of this detection mechanism to detect low impact damages in a quick and keen way by using area detection. The purpose of this work was to find these damaged spots and provide position information for further damage analysis, which may gather more information about the damage profile than thermography analysis does. This could be, for example, an ultrasonic-based detection mechanism, the main disadvantage of which is the lack of a quick possibility to spot barely or no visible damages in large areas, because of the single-point detection sensors. The following conclusions can be made from the tests:

- For lower impact energies (up to 3 J), impact events were elastic, and excessive impact energy was used for the rebounding of the impactor. As the damaged specimens were visually inspected, maybe, minor matrix cracking or impactor signs were observed on the carbon surface of the impacted side of the specimens. However, the thermography analysis showed, in a quick way, that there was delamination between the inner layers of the carbon specimen. Due to that fact, further examinations on that part should be done.
- At increasing impact energies (approximately 4 J), the damage profile is in a transition, where damage detection with visual inspection was possible, and the F/t impact curves gave further information about possible damage. In that case, thermography analysis can give more detailed information, but direct use of ultrasonic analysis would be more efficient, because the damages were already spotted by the visual inspection.
- Impact energies around 5 J and above will cause catastrophic damage to the testing specimen, and for inspection, the visual methods were the fastest way and accurate enough.
- The non-destructive thermography testing method showed its potential, especially at lower impact energies because it is a quick, keen and non-destructive area detection method. A possible field of operation is the inspection of large carbon parts in automotive and/or airplane applications.
